# Therapeutic activity of two xanthones in a xenograft murine model of human chronic lymphocytic leukemia

**DOI:** 10.1186/1756-8722-3-49

**Published:** 2010-12-07

**Authors:** Séverine Loisel, Karine Le Ster, Michèle Meyer, Christian Berthou, Pierre Youinou, Jean-Pierre Kolb, Christian Billard

**Affiliations:** 1 EA 2216, Université de Bretagne, Brest, France; 2 Centre Hospitalier Universitaire, Brest, France; 3 USM502-UMP5154 CNRS, Muséum National d'Histoire Naturelle, Paris, France; 4 INSERM U872, Equipe 18, Centre de Recherche des Cordeliers, Paris, France; 5 Université Pierre et Marie Curie UMRS 872, Paris, France; 6 Université Paris Descartes UMRS 872, Paris, France

## Abstract

**Background:**

We previously reported that allanxanthone C and macluraxanthone, two xanthones purified from *Guttiferae *trees, display *in vitro *antiproliferative and proapoptotic activities in leukemic cells from chronic lymphocytic leukemia (CLL) and leukemia B cell lines.

**Results:**

Here, we investigated the *in vivo *therapeutic effects of the two xanthones in a xenograft murine model of human CLL, developed by engrafting CD5-transfected chronic leukemia B cells into SCID mice. Treatment of the animals with five daily injections of either allanxanthone C or macluraxanthone resulted in a significant prolongation of their survival as compared to control animals injected with the solvent alone (*p *= 0.0006 and *p *= 0.0141, respectively). The same treatment of mice which were not xenografted induced no mortality.

**Conclusion:**

These data show for the first time the *in vivo *antileukemic activities of two plant-derived xanthones, and confirm their potential interest for CLL therapy.

## 

To the Editor,

Despite recent therapeutic advances with the combination of purine analogs, alkylating agents and monoclonal antibodies, chronic lymphocytic leukemia (CLL) remains an incurable disease [1-3]. It is characterized by the clonal expansion of a population of CD5^+ ^B lymphocytes and by the accumulation in the blood of leukemic cells that are quiescent but defective in their apoptotic program [2,4]. Thus, CLL is a disease of proliferation as well as accumulation. Treatments targeting both dividing and apoptosis-deficient quiescent cells might therefore improve the CLL patients' outcome [2-4]. A number of plant-derived compounds were found to exhibit *in vitro *capacities to either inhibit leukemic cell growth or induce apoptosis or both, but their clinical use was hampered by the lack of *in vivo *studies on animal models of CLL. However, some murine models recapitulating the human CLL disease were described lately, such as the *TCL1 *transgenic mouse model developing a CD5+ B cell lymphoproliferative disease typical of aggressive CLL [5]. We previously showed that several xanthones purified from african trees of the Guttiferae family display both antiproliferative and proapoptotic properties in cell lines derived from CLL and hairy cell leukemia (HCL), another chronic B-cell leukemia [6]. In addition, these compounds can induce the apoptosis of primary CLL cells *in vitro *through different mechanisms [6]. It seemed therefore crucial to determine whether some xanthones are capable of *in vivo *therapeutic effects in an animal model of CLL.

We selected two of the xanthones which were purified and characterized in our previous study [6] on the basis of their *in vitro *activities in CLL cells and their hardly detectable toxicity in B lymphocytes from healthy donors: (i) allanxanthone C, a xanthenedione that we have identified as acting by caspase activation, possibly through a mechanism involving inhibition of the NO pathway [4]; and (ii) macluraxanthone, originaly found to inhibit the growth of solid tumor cell lines [7] and moreover, capable of triggering the mitochondrial pathway of apoptosis in CLL cells [6]. Taking advantage of our previous data [8], we developed a xenograft mouse model by engrafting CD5-transfected human JOK-1 cells into SCID mice (Le Ster *et al*, submitted). Actually, it was demonstrated that transplantation of this cell line JOK-1 into SCID mice led to the establishment of a CLL model, allowing the evaluation of the antileukemic efficacy of fludarabine phosphate [9]. Furthermore, we reported that CD5 plays a prominent role in the control of CLL cell apoptosis through its distribution in lipid rafts and its interaction with the B-cell receptor [10]. Whereas CD5 is generally lost in long-term cultures of CLL cell lines, JOK-1/5.3 cells derived by stable transfection of the human CD5 gene into JOK-1 cells display a phenotype somewhat close to that of primary leukemic cells. The xenografted mice that we obtained developed a leukemia resembling the CLL type as defined by the French-American-British criteria.

We first verified that the xanthones were active on the JOK-1/5.3 cells used for engrafting the mice. Treatment with either allanxanthone C or macluraxanthone for 18 h resulted in a concentration-dependent inhibition of cell growth, peaking at respectively 40% and 70% with 40 μM (estimated by ^3^H-thymidine uptake), in accordance with our previous data on CLL and HCL cell lines [6]. Both compounds induced the accumulation in the G_0_/G_1 _phase of the cell cycle as compared to untreated cells (*P *< 0.05) and decreased the percentages of cells in S and G_2_/M phases (evaluated by propidium iodide incorporation using flow cytometry and Multicycle AV program). Two other xanthones, 1,7-dihydroxanthone and α-mangostin which were inactive in our previous study [6] were used as negative controls. The proapoptotic capacities of allanxanthone C and macluraxanthone were also checked in JOK-1/5.3 cells by stimulation of phosphatidylserine externalization (quantified by annexin V-FITC binding), although these cells turned out to be less sensitive than primary CLL cells.

For the *in vivo *experiments, randomised groups of SCID CB-17 mice were inoculated with 10^7 ^JOK-1/5.3 cells (day 0). Xenografted mice were treated at days 3 to 7 with five daily injections of either allanxanthone C or macluraxanthone (5 mg/kg) or solvent alone as untreated control. The three groups of mice were then monitored daily and the survival was estimated according to the Kaplan-Meier's method (Figure 1). Mean survival times ± SE were 25.6 ± 0.6 days and 26.0 ± 1.7 days for respectively allanxanthone C and macluraxanthone-treated mice *versus *20.2 ± 0.8 days for untreated control mice. These increases in survival (27% and 29% respectively) were significant with *P *values of 0.0006 for allanxanthone C group and of 0.0141 for macluraxanthone group as compared to control group (according to the Student's unpaired t-test). No significant difference was detected between the two groups of xanthone-treated mice (*P *= 0.83). These results show that treatments of the xenografted mice with allanxanthone C and macluraxanthone resulted in a prolongation of their lifespan.

**Figure 1:**
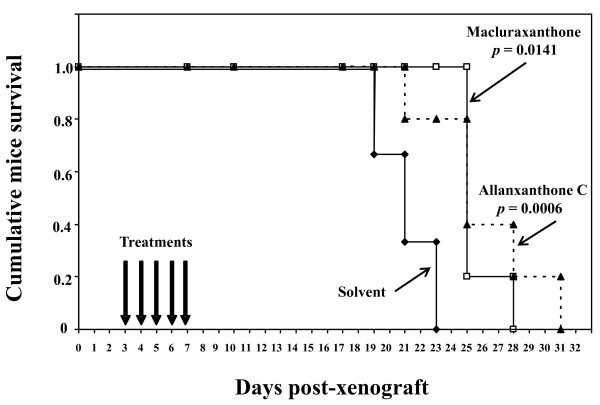
**Xanthones prolong the survival of SCID mice xenografted with human JOK1/5.3 cells**. Three randomized groups of 6-8 weeks old female CB-17 SCID mice (5/group) were inoculated intravenously with 10 × 10^6 ^JOK-1/5.3 cells (in 0.1 ml PBS) on day 0 and treated with 5 daily iv injections (0.2 ml in saline) on days 3 to 7 (arrows) of 5 mg/kg of allanxanthone C (black triangle) or macluraxanthone in DMSO (white square) or of solvent (DMSO in saline) alone (black losange). The three groups of xenografted mice were then checked daily for survival and the cumulative survival data were analyzed according to the Kaplan-Meier's curves. For details, see the text.

To check a toxicity of the xanthones, two groups of 5 mice which were not inoculated with JOK-1/5.3 cells were treated with either allanxanthone C or macluraxanthone according to the same protocol as before. No lethality was observed in these two groups of animals, suggesting an absence of toxicity of the xanthones *per se *under the treatment protocol used. This also favors that the deaths observed in the JOK-1/5.3-grafted mice were due to the presence of the leukemic cells, and that treatments with the xanthones were able to delay significantly these lethal effects.

In conclusion, results presented in this letter show for the first time that allanxanthone C and macluraxanthone purified from Guttiferaes are capable of *in vivo *antileukemic effects in a xenograft murine model of human CLL. These therapeutic activities of the natural compounds, of similar extent, occur without apparent toxicity. Although the comparison with known chemotherapeutic agents has to be performed, our data provide further confirmation that these xanthones might be used as new agents for the therapy of CLL and possibly allied chronic B cell malignancies. Experiments examining the effects of increasing doses and time of treatment as well as different schedules of administration are in progress in order to improve the therapeutic efficacy of the two xanthones. Studies of their exact mechanisms of action in primary CLL patients' cells are also considered in order to define therapeutic targets.

## Competing interests

The authors declare that they have no competing interests.

## Authors' contributions

SV performed in vivo studies, analyzed the data and revised the manuscript; KLS performed *in vitro *experiments; MM purified the xanthones. CBe contributed to design the study; PY designed the study; JPK designed the study, interpreted the data and revised the manuscript; CBi interpreted the data and wrote the manuscript. All authors read and approved the final manuscript.
